# Social networking site use, depressive and anxiety symptoms in adolescents: evidence from a longitudinal cohort study (SCAMP)

**DOI:** 10.1186/s12916-026-04667-5

**Published:** 2026-02-03

**Authors:** Chen Shen, Braulio M. Girela-Serrano, Martina Di Simplicio, Alexander Spiers, Iroise Dumontheil, Michael S. C. Thomas, Martin Röösli, Paul Elliott, Rachel B. Smith, Mireille B. Toledano

**Affiliations:** 1 Mohn Centre for Children’s Health and Wellbeing, School of Public Health, Imperial College London, 90 Wood Lane, London, W12 0BZ UK; 2 National Institute for Health Research Health Protection Research Unit in Radiation Threats and Hazards, Imperial College London, London, UK; 3 Division of Psychiatry, Department of Brain Sciences, Imperial College London, London, UK; 4 Child and Adolescent Mental Health Services, Central North West London NHS Foundation Trust, London, UK; 5 Centre for Educational Neuroscience, Department of Psychological Sciences, Birkbeck, University of London, London, UK; 6 Melbourne School of Psychological Sciences, The University of Melbourne, Melbourne, Australia; 7 Department of Epidemiology and Public Health, Swiss Tropical and Public Health Institute, Basel, 4051 Switzerland; 8 University of Basel, Basel, Switzerland; 9 NIHR Biomedical Research Centre, Imperial College London, London, UK

**Keywords:** Social network site, Depressive symptoms, Anxiety symptoms, Sleep, Adolescent, Longitudinal cohort study, Mediation analysis

## Abstract

**Background:**

The growing and pervasive use of social network sites (SNS) has raised concerns about their impact on adolescent mental health during this sensitive developmental phase. Existing longitudinal studies are constrained by methodological limitations and limited exploration of underlying mechanisms. We investigated the longitudinal associations between SNS use and depressive and anxiety symptoms in adolescents and whether sleep mediated these associations.

**Methods:**

We analysed longitudinal data from 2350 adolescents from 31 schools in London, participating in the Study of Cognition, Adolescents, and Mobile Phones (SCAMP). The exposure was self-reported duration of SNS use at baseline (aged 11–12 years). Outcomes were depressive and anxiety symptoms at follow-up, analysed as symptom severity and clinically significant symptoms (aged 13–15 years). The associations between SNS use and depressive and anxiety symptoms were assessed via multi-level ordinal logistic regression (symptom severity) and logistic regression (clinically significant symptoms). The mediation effects of insufficient sleep, sleep onset latency, and sleep disturbance were assessed by mediation analysis.

**Results:**

Compared to 0–30 min per day, more than 3 h per day of SNS use at baseline was associated with higher severity levels of depressive and anxiety symptoms (adjusted odds ratio (OR) = 1.47, 95% CI 1.12, 1.93 and OR = 1.40, 95% CI 1.06, 1.83, respectively) and clinically significant depressive and anxiety symptoms at follow-up (OR = 1.70, 95% CI 1.19, 2.42 and OR = 1.60, 95% CI 1.11, 2.31, respectively). The associations between total and weekend SNS use and depressive symptom severity were stronger in girls than boys. Other associations were similar by gender. Insufficient sleep duration (particularly on weekdays) and sleep onset latency at baseline partly mediated the associations of SNS use and depressive and anxiety symptoms (proportion of mediation ranged between 11.1% and 33.1%). The mediation effects of sleep disturbance were less marked.

**Conclusions:**

In a large longitudinal cohort, we found that SNS use exceeding 3 h per day is associated with increased risks of depressive and anxiety symptoms in adolescents. Findings from mediation analysis suggest that addressing poor sleep hygiene in relation to SNS use might mitigate the negative impact of high SNS use. Our findings may inform the development of early secondary school curricula incorporating digital literacy and sleep hygiene education.

**Supplementary Information:**

The online version contains supplementary material available at 10.1186/s12916-026-04667-5.

## Background

Adolescence is a life stage in which individuals are vulnerable to the onset of mental illness. It has been estimated that half of all lifetime mental disorders are developed by age 14 years [1, 2]. Of these, depressive and anxiety disorders are the most common child and adolescent mental disorders both in the UK and worldwide, with a higher prevalence in girls than boys [3]. Depression and anxiety are associated with higher risks of suicide and substance use, and have adverse impacts on social functioning, educational achievement, and economic potential [4–7].

Adolescence is a dynamic developmental period of widespread social and emotional transformation, characterised by a change in the intensity and quality of communication between peers and close friends. This is reflected in a transition from family-centred life into a context where peers exert a significant influence with an amplified value of peer approval and rejection [8]. Together with the continued maturation of cognitive skills, this allows adolescents to become independent at navigating social contexts and contributes to the development of self-identity and regulation abilities that lead into adulthood [9]. As part of this process, social network sites (SNS) provide a medium to multiply connections, build meaningful relationships, and foster a sense of belonging. However, SNS use also introduces negative experiences such as online harassment, cyberbullying, and dissatisfaction with body image, which are more prevalent and impactful in girls [10].

There has been extensive research on SNS use and mental health in young people. Recent systematic reviews and meta-analyses have reported positive associations between SNS use and mental health problems, albeit with high heterogeneity across studies [11–14]. Common methodological limitations include cross-sectional designs, inadequate SNS measurements (e.g. lacking device-specific, weekday/weekend distinctions, or capturing frequency without duration), small sample sizes, and the absence of moderation analyses. Several longitudinal studies have addressed some of these limitations by virtue of large sample sizes and gender-specific analyses [15–19], but none of these studies has detailed measures of SNS use. Some mental health measures (e.g. internalising difficulties) do not have clinical implications. To advance existing evidence with a more clinical focus and directly address an important public health concern, it is needed to examine depressive and anxiety symptoms in relation to SNS use. It has been shown a potential threshold effect of SNS use on mental wellbeing (i.e. moderate use is not detrimental) [20]. However, such threshold patterns have not been explicitly investigated for depressive or anxiety symptoms, where evidence could inform targeted intervention. Moreover, no longitudinal studies have explored the potential underlying mechanisms linking SNS use duration and depressive and anxiety symptoms. Such mechanistic evidence strengthens causal inference and enables a better understanding of how SNS use impacts depressive and anxiety symptoms.

Adolescence is also characterised by a high prevalence of sleep deficits and delayed bedtime, due to both biological maturation and environmental factors [21]. SNS use, and particularly nighttime use, has been associated with insufficient sleep and poor sleep quality [22]. Conversely, sleep problems are well-documented risk factors for poor mental health in adolescents [23, 24]. Thus, sleep might play an important role in the pathway between SNS use and mental health symptoms.

We leveraged longitudinal data on digital technology use, sleep, and mental health from the Study of Cognition, Adolescents, and Mobile Phones (SCAMP), a large-scale adolescent cohort study across Greater London [25]. In the present study, we aimed to assess longitudinal associations between SNS use and depressive and anxiety symptoms, including potential effect modification by gender. We also examined whether sleep problems mediated these associations.

## Methods

### Participants

SCAMP is the world’s largest prospective adolescent cohort study dedicated to investigating the use of mobile phones and other wireless technologies in relation to cognitive, behavioural, and mental health outcomes. Details of this study have been reported elsewhere [25]. Between November 2014 and July 2016, baseline data were collected from 6590 participants in year 7 (aged 11–12 years) from 39 secondary schools (26 state/13 independent) across Greater London, UK. Of these participants, 3814, from 31 schools, participated in a follow-up assessment between November 2016 and July 2018 when they were in year 9/10 (aged 13–15 years). Attrition at follow-up was primarily due to school drop-out, participant opt-out and withdrawal, and absence at the time of assessment. Participants in all SCAMP schools completed a computer-based assessment via the Psytools software (Delosis Ltd, London), in examination conditions. The assessment included a detailed questionnaire on their digital technology behaviours (e.g. smartphone use, SNS use, and video gaming), mental health and behavioural scales, a battery of cognitive tests, lifestyle, and health. Participants with complete data on SNS use, confounders, and depressive or anxiety symptoms were included in the present analysis (*n* = 2316 for depressive symptoms; *n* = 2350 for anxiety symptoms).

### Measures

#### Exposure: SNS use at baseline

We examined self-reported duration of SNS use on mobile phones and other devices, via the questions: “How much time per day do you spend on social network sites (e.g. Facebook, Instagram, Twitter) on your mobile phones?” and “How much time per day do you spend on social network sites (e.g. Facebook, Instagram, Twitter) on other devices (do not include mobile phones)?”. Weekday and weekend use were assessed separately for both mobile phones and other devices. Participants were provided with the following response options: 0 min; 1–10 min; 11–30 min; 31–59 min; 1–2 h; 3–4 h; more than 5 h. Daily duration of total SNS use (weighted average of weekday and weekend use), weekday use, and weekend use were included as exposure variables. These exposure variables were derived by summing the duration of SNS use on mobile phones and other devices, taking the midpoints of the response category intervals, except for the highest category where we used the category lower bound (i.e. 5 h/day). Exposure variables were analysed both as categorical variables (0–30 min (reference category), 31–59 min, 1–2 h, and 3 h +) and continuous variables. For analyses using continuous variables, we calculated the interquartile range (IQR) of each exposure variable and examined the associations between per one-IQR increase in SNS use and depressive and anxiety symptoms.

#### Outcome: depressive and anxiety symptoms at follow-up

Depressive symptoms were assessed using the 9-item Patient Health Questionnaire (PHQ-9), with a higher score indicating more depressive symptoms. Depressive symptom severity was categorised using the total PHQ-9 score as follows: no or minimal (0–4), mild (5–10), moderate (11-14), and moderately severe or severe (15 +). A summary score of 11 or above is considered indicative of clinically significant depressive symptoms [26]. Anxiety symptoms were assessed using the 7-item Generalised Anxiety Disorder (GAD-7) scale, with a higher score indicating more anxiety symptoms. Anxiety symptom severity was categorised using the total GAD-7 score as follows: no or minimal (0–4), mild (5–9), moderate (10–14), and moderately severe or severe (15 +). A summary score of 10 or above is considered indicative of clinically significant anxiety symptoms [27]. Among adolescents, both PHQ-9 and GAD-7 demonstrate good sensitivity and specificity for detecting clinically significant symptoms [26, 28].

#### Mediator: insufficient sleep duration, sleep onset latency (SOL), and sleep disturbance at baseline

Sleep duration at baseline was derived from self-reported bedtime, SOL, and wake time, specified by weekdays and weekends. Details have been reported elsewhere [23]. Insufficient sleep was classified as sleep duration less than 9 h based on the recommendations of the National Sleep Foundation [29]. Sleep quality was measured using a 4-item sleep disturbance scale [30]. Participants reported how often they experienced difficulty falling asleep, restless sleep, waking during the night, and early morning awakening, using a four-point Likert scale (never, rarely, sometimes, often). Total score ranged from 0 to 12, with a higher score indicating greater sleep disturbance.

#### Covariates

Demographic information including age, gender, ethnicity, and parental occupation was captured in the SCAMP assessment. Ethnicity was categorised as White, Black, Asian, and mixed/others. Parental socioeconomic status (SES) was derived according to the parental occupation, categorising into three levels (managerial and professional, intermediate, and routine or manual) based on the National Statistics Socio-economic classification (NS-SEC) [31]. If these differed between parents, participants were assigned the higher NS-SEC level. We also included baseline scores of Strengths and Difficulties Questionnaire (SDQ) emotional and peer problem subscales, together forming the internalising subscale with scores ranging from 0 to 20 [32]. A summary score of 10 or above of the internalising subscale was defined as the presence of internalising difficulties (including borderline) [33]. At baseline, participants were asked three questions on the frequency of substance use (alcohol, tobacco, marijuana) in the last 6 months.

### Statistical analysis

#### Main analysis

Multi-level ordinal logistic regression was used to assess the associations between baseline SNS use across all devices and severity of depressive and anxiety symptoms at follow-up. Multi-level logistic regression was used to assess the associations between baseline SNS use and the presence of clinically significant depressive and anxiety symptoms at follow-up. Potential confounders included age, gender, ethnicity, parental SES, and baseline score of SDQ internalising subscale, selected a priori. We also examined whether these associations varied by gender by testing the significance of the interaction term and stratified the associations by gender.

Mediation analysis was performed to test the hypothesis that insufficient sleep duration, SOL, and sleep disturbance might be mediators on the pathway between SNS use and subsequent depressive and anxiety symptoms [34]. To investigate the mediation effects of each sleep variable and potential differential mediation effects between weekday and weekend sleep problems, we first examined the individual mediation effects of weekday insufficient sleep duration and SOL on weekday SNS use and depressive and anxiety symptoms. We then repeated these analyses for weekend sleep measures using weekend SNS use as the exposure. Mediation effects of sleep disturbance on total SNS use and outcomes were examined. To account for the inter-relationships among these sleep variables, we also examined the joint effects of sleep problems on total SNS use and depressive and anxiety symptoms by including insufficient sleep, SOL, and sleep disturbance within a single model.

The Karlson-Holm-Breen method was used to decompose the total effect into direct and indirect effects by comparing coefficients across nested non-linear probability models including ordinal logistic regression and logistic regression [35]. Mediation analysis based on these non-linear models is subject to non-collapsibility bias [36] when additional covariates (i.e. potential mediators) are introduced. This means the differences between the total effect (without mediators) and the direct effect (with mediators) may not only reflect the mediation effect but also the non-collapsibility effect. The Karlson-Holm-Breen method controls for this non-collapsibility effect, thereby providing unbiased estimates of mediation effects.

#### Sensitivity analysis

We performed a series of sensitivity analyses (SA) to confirm the robustness of our findings. SA1: We excluded participants with baseline internalising difficulties to assess the associations between SNS use and later depressive and anxiety symptoms in participants without pre-existing emotional and peer problems. SA2: We further adjusted for substance use variables. Due to the high proportion of missingness in these variables (17% for each variable in the analytical sample), we treated missingness as a special category rather than excluding participants who did not respond to these questions. Substance use variables were categorised as never use, ever use, and missing. Therefore, sample sizes remained unchanged after additional adjustment for substance use variables. SA3: To minimise potential biases due to missing covariates, we used multiple imputation in participants with complete exposure and outcome variables to predict missing ethnicity (0.6%), parental SES (2.5%), and internalising difficulties (7.1%) based on a flexible additive regression model, incorporating data on weekday and weekend SNS use, scores of PHQ-9, GAD-7, gender, age, and school type. We imputed 20 datasets and then summarised the results from 20 imputed datasets into single estimates with 95% CIs adjusted for missing data uncertainty. SA4: We also examined the associations of baseline SNS use on mobile phones and other devices with follow-up depressive and anxiety symptoms to determine if patterns of association differed across devices. All analyses were performed using STATA (version 16.1) and R (version 4.4.2).

## Results

The analytical sample was diverse in terms of socio-demographic characteristics (Table 1). At baseline, the median use of total SNS per day was 0.34 h (~ 20 min), with higher use on weekends (median 0.43 h) than weekdays (0.34 h). Follow-up total SNS use (for descriptive purposes only) increased to 1.13 h (median). Insufficient sleep at baseline was more prevalent on weekdays than weekends. The prevalence of clinically significant depressive and anxiety symptoms at follow-up was 14.3% (331/2316) and 13.1% (309/2350), respectively. Participants in the analytical sample had a higher proportion of girls, Asian participants, and independent school students, and reported lower SNS use than baseline-only participants (Additional file 1: Table S1). Parental SES was not significantly different between the analytical sample and baseline-only sample.

**Table 1:** Descriptive statistics of the analytical sample

Socio-demographic variables	Depressive (*n* = 2316)	Anxiety (*n* = 2350)
Age (years) at baseline, mean (SD)	11.59 (0.47)	11.59 (0.47)
Age (years) at follow-up, mean (SD)	13.88 (0.53)	13.87 (0.53)
Gender, *n* (%)		
Male	1044 (45.1)	1058 (45.0)
Female	1272 (54.9)	1292 (55.0)
Ethnicity, *n* (%)		
White	993 (42.9)	1006 (42.8)
Black	244 (10.5)	250 (10.6)
Asian	774 (33.4)	781 (33.2)
Mixed/others	305 (13.2)	313 (13.3)
Parental socioeconomic status^a^, *n* (%)		
Managerial/professional occupations	1373 (59.3)	1389 (59.1)
Intermediate occupations	503 (21.7)	511 (21.7)
Routine and manual occupations	440 (19.0)	450 (19.2)
Type of school, *n* (%)		
Independent	684 (29.5)	687 (29.2)
State	1632 (70.5)	1663 (70.8)
Substance use variables (baseline)		
Alcohol, *n* (%)		
Never use	1677 (72.4)	1698 (72.3)
Ever use	247 (10.7)	250 (10.6)
Missing	392 (16.9)	402 (17.1)
Tobacco, *n* (%)		
Never use	1874 (80.9)	1898 (80.8)
Ever use	50 (2.2)	50 (2.1)
Missing	392 (16.9)	402 (17.1)
Marijuana, *n* (%)		
Never use	1894 (81.8)	1918 (81.6)
Ever use	30 (1.3)	30 (1.3)
Missing	392 (16.9)	402 (17.1)
Exposures (baseline, across all devices)		
Total SNS use (h), median (IQR)	0.34 (0.03, 1.24)	0.34 (0.03, 1.23)
Weekday average SNS use (h), median (IQR)	0.34 (0, 1.09)	0.34 (0, 1.09)
Weekend average SNS use (h), median (IQR)	0.43 (0.05, 1.5)	0.43 (0.09, 1.5)
Exposures (follow-up^b^, across all devices)		
Total SNS use (h), median (IQR)	1.13 (0.34, 3.04)	1.13 (0.36, 3.04)
Weekday average SNS use (h), median (IQR)	0.84 (0.34, 2.25)	0.84 (0.34, 2.25)
Weekend average SNS use (h), median (IQR)	1.5 (0.43, 3.84)	1.5 (0.43, 3.84)
Mediators (baseline)		
Insufficient weekday sleep^c^, *n* (%)	1114 (48.1)	1130 (48.1)
Insufficient weekend sleep, *n* (%)	665 (28.7)	673 (28.6)
Weekday SOL (h), median (IQR)	0.25 (0.25, 0.5)	0.25 (0.25, 0.5)
Weekend SOL (h), median (IQR)	0.25 (0.08, 0.75)	0.25 (0.08, 0.75)
Sleep disturbance (0–12), median (IQR)	5 (3, 7)	5 (3, 7)
Outcomes (follow-up)		
Symptom severity, *n* (%)		
No or minimal	1393 (60.2)	1535 (65.3)
Mild	592 (25.6)	506 (21.5)
Moderate	159 (6.9)	187 (8.0)
Moderately severe or severe	172 (7.4)	122 (5.2)
Presence of clinically significant symptoms, *n* (%)	331 (14.3)	309 (13.1)

*IQR *interquartile range, *SNS *social networking site, *SOL *sleep onset latency

^a^Parental socioeconomic status was derived based on National Statistics Socio-economic classification of occupation

^b^For descriptive purposes only

^c^Insufficient sleep was defined as sleep duration less than 9 h

Positive correlations between SNS use and PHQ-9 and GAD-7 scores were found (Additional file 1: Fig. S1). Table 2 shows that compared to the lowest SNS usage category (i.e. 0–30 min per day), only the highest total SNS usage category (i.e. more than 3 h/day) was significantly associated with higher depressive (OR = 1.47, 95% CI 1.12, 1.93) and anxiety (OR = 1.40, 95% CI 1.06, 1.83) symptom severity levels at 2-year follow-up, after adjusting for age, gender, ethnicity, parental SES, baseline score of SDQ internalising subscale, and school clustering effects. Per IQR increase in total SNS use was also associated with higher depressive and anxiety symptom severity levels. Similar patterns of association were observed for weekday and weekend SNS use.

**Table 2:** Associations between baseline SNS use across all devices and depressive and anxiety symptom severity levels at 2-year follow-up using ordinal logistic regression

Symptom severity	SNS use	Daily average duration	*N*	Unadjusted model OR (95% CI)	Adjusted model^a^ OR (95% CI)
Depressive	Total use	0–30 min	1346	1	1
		31–59 min	311	1.23 (0.97, 1.57)	1.21 (0.94, 1.55)
		1–2 h	385	1.14 (0.91, 1.43)	1.12 (0.89, 1.42)
		3 h +	274	**1.80 (1.39, 2.31)**	**1.47 (1.12, 1.93)**
	Per IQR increase	1.31 h	2316	**1.14 (1.09, 1.20)**	**1.10 (1.04, 1.16)**
	Weekday use	0–30 min	1473	1	1
		31–59 min	250	1.15 (0.89, 1.49)	1.19 (0.91, 1.57)
		1–2 h	346	1.15 (0.92, 1.45)	1.13 (0.89, 1.44)
		3 h +	247	**1.81 (1.39, 2.35)**	**1.45 (1.10, 1.92)**
	Per IQR increase	1.09 h	2316	**1.11 (1.07, 1.16)**	**1.08 (1.03, 1.13)**
	Weekend use	0–30 min	1241	1	1
		31–59 min	276	0.90 (0.69, 1.18)	0.88 (0.67, 1.17)
		1–2 h	368	**1.32 (1.05, 1.65)**	1.25 (0.99, 1.58)
		3 h +	431	**1.46 (1.17, 1.81)**	1.22 (0.97, 1.54)
	Per IQR increase	1.5 h	2316	**1.14 (1.09, 1.19)**	**1.10 (1.05, 1.16)**
Anxiety	Total use	0–30 min	1365	1	1
		31–59 min	316	1.06 (0.83, 1.37)	1.04 (0.79, 1.35)
		1–2 h	390	1.11 (0.88, 1.40)	1.07 (0.84, 1.37)
		3 h +	279	**1.82 (1.41, 2.35)**	**1.40 (1.06, 1.83)**
	Per IQR increase	1.31 h	2350	**1.16 (1.11, 1.22)**	**1.10 (1.05, 1.16)**
	Weekday use	0–30 min	1497	1	1
		31–59 min	253	0.80 (0.60, 1.06)	0.81 (0.60, 1.09)
		1–2 h	348	1.14 (0.90, 1.45)	1.08 (0.84, 1.39)
		3 h +	252	**1.77 (1.36, 2.30)**	**1.34 (1.01, 1.77)**
	Per IQR increase	1.09 h	2350	**1.13 (1.08, 1.18)**	**1.08 (1.03, 1.13)**
	Weekend use	0–30 min	1254	1	1
		31–59 min	285	0.96 (0.73, 1.26)	0.95 (0.71, 1.26)
		1–2 h	373	1.09 (0.86, 1.38)	0.99 (0.77, 1.27)
		3 h +	438	**1.62 (1.31, 2.01)**	**1.30 (1.03, 1.64)**
	Per IQR increase	1.5 h	2350	**1.16 (1.10, 1.21)**	**1.10 (1.05, 1.16)**

*SNS *social networking site, *OR *odds ratio, *IQR *interquartile range

^a^Adjusted for age, gender, ethnicity, parental socioeconomic status, baseline score of Strengths and Difficulties Questionnaire internalising subscale, and school clustering effect

Depressive and anxiety symptom severity levels (4 levels): no or minimal, mild, moderate, and moderately severe or severe

OR in ordinal logistic regression reflects the change in the odds of being in a higher category of the outcome variable (e.g. depressive and anxiety symptom severity levels) associated with a one-unit increase in the exposure variable (e.g. total SNS use)

Boldface indicates statistically significant associations (*P*<0.05)

Table 3 shows a similar threshold pattern in the associations between SNS use and clinically significant depressive and anxiety symptoms, with only the highest total SNS use category (more than 3 h/day) significantly associated with these outcomes (depressive: OR = 1.70, 95% CI 1.19, 2.42; anxiety: OR = 1.60, 95% CI 1.11, 2.31). Per IQR increase in total SNS use was also associated with clinically significant depressive and anxiety symptoms. Weekday and weekend SNS use (more than 3 h/day and per IQR increase) were associated with clinically significant depressive and anxiety symptoms.

**Table 3:** Associations between baseline SNS use across all devices and clinically significant depressive and anxiety symptoms at 2-year follow-up

Clinically significant symptoms	SNS use	Daily average duration	*N*	Unadjusted model OR (95% CI)	Adjusted model^a^ OR (95% CI)
Depressive	Total use	0–30 min/day	1346	1	1
		31–59 min/day	311	1.03 (0.71, 1.48)	0.99 (0.68, 1.46)
		1–2 h/day	385	1.08 (0.78, 1.51)	1.10 (0.78, 1.55)
		3 h +/day	274	**2.07 (1.50, 2.85)**	**1.70 (1.19, 2.42)**
	Per IQR increase	1.31 h	2316	**1.19 (1.12, 1.26)**	**1.16 (1.09, 1.24)**
	Weekday use	0–30 min/day	1473	1	1
		31–59 min/day	250	0.99 (0.66, 1.48)	1.05 (0.69, 1.59)
		1–2 h/day	346	1.06 (0.75, 1.50)	1.05 (0.73, 1.51)
		3 h +/day	247	**2.26 (1.63, 3.13)**	**1.88 (1.31, 2.68)**
	Per IQR increase	1.09 h	2316	**1.15 (1.10, 1.21)**	**1.13 (1.07, 1.19)**
	Weekend use	0–30 min/day	1241	1	1
		31–59 min/day	276	0.96 (0.65, 1.43)	0.96 (0.64, 1.46)
		1–2 h/day	368	1.08 (0.77, 1.52)	1.02 (0.71, 1.46)
		3 h +/day	431	**1.78 (1.34, 2.38)**	**1.50 (1.10, 2.05)**
	Per IQR increase	1.5 h	2316	**1.19 (1.13, 1.26)**	**1.16 (1.09, 1.23)**
Anxiety	Total use	0–30 min/day	1365	1	1
		31–59 min/day	316	1.14 (0.79, 1.64)	1.05 (0.71, 1.56)
		1–2 h/day	390	1.10 (0.78, 1.55)	1.07 (0.74, 1.53)
		3 h +/day	279	**2.14 (1.54, 2.97)**	**1.60 (1.11, 2.31)**
	Per IQR increase	1.31 h	2350	**1.19 (1.12, 1.26)**	**1.13 (1.06, 1.21)**
	Weekday use	0–30 min/day	1497	1	1
		31–59 min/day	253	0.66 (0.41, 1.06)	0.65 (0.40, 1.07)
		1–2 h/day	348	1.28 (0.92, 1.78)	1.21 (0.85, 1.73)
		3 h +/day	252	**2.03 (1.45, 2.84)**	**1.52 (1.04, 2.21)**
	Per IQR increase	1.09 h	2350	**1.15 (1.10, 1.21)**	**1.11 (1.05, 1.18)**
	Weekend use	0–30 min/day	1254	1	1
		31–59 min/day	285	1.10 (0.74, 1.62)	1.01 (0.67, 1.53)
		1–2 h/day	373	0.99 (0.69, 1.42)	0.87 (0.59, 1.27)
		3 h +/day	438	**1.80 (1.34, 2.42)**	**1.38 (1.00, 1.90)**
	Per IQR increase	1.5 h	2350	**1.18 (1.11, 1.25)**	**1.12 (1.05, 1.20)**

*SNS *social networking site, *OR *odds ratio, *IQR *interquartile range

^a^Adjusted for age, gender, ethnicity, parental socioeconomic status, baseline score of Strengths and Difficulties Questionnaire internalising subscale, and school clustering effect

Boldface indicates statistically significant associations (P<0.05)

**Fig. 1:**
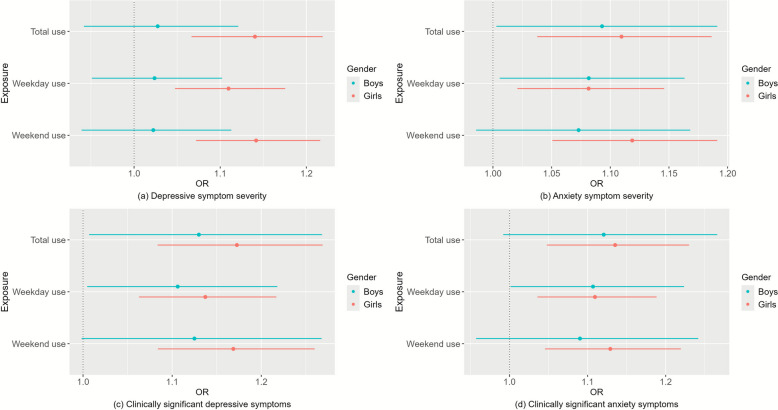
Gender-specific associations between per IQR increase in SNS use across all devices and depressive and anxiety symptoms

The associations between total and weekend SNS use and depressive symptom severity were stronger in girls than boys (*P* values for gender interaction < 0.05). Other associations were similar by gender. Stratified analyses by gender are shown in Fig. 1.

Table 4 shows that weekday insufficient sleep, but not weekend, partly mediated the associations between per IQR increase in SNS use and depressive and anxiety symptom severity levels (mediation proportion 22.0% and 11.1%, respectively). Both weekday and weekend SOL also partly mediated the associations of SNS use with depressive and anxiety symptom severity levels (mediation proportion ranged between 19.7% and 33.1%). Compared to insufficient sleep and SOL, sleep disturbance played a relatively weaker role in mediating the associations. Combined, sleep problems mediated 49.6% and 32.4% of the associations between total SNS use and depressive and anxiety symptom severity levels, respectively.

**Table 4:** Mediation effects of sleep on the associations between SNS use across all devices and depressive (*n* = 2316) and anxiety (*n* = 2350) symptom severity levels

Symptom severity	SNS use (per IQR increase)	Sleep measures (mediator)	Indirect effect *b* (95% CI)	Direct effect *b* (95% CI)	Total effect *b* (95% CI)	Mediation proportion (%)
Depressive	Weekday use	Insufficient weekday sleep	0.02 (0.01, 0.02)	0.06 (0.01, 0.10)	0.07 (0.03, 0.12)	22.0
	Weekend use	Insufficient weekend sleep	0.01 (− 0.00, 0.02)	0.08 (0.03, 0.13)	0.09 (0.04, 0.14)	–
	Weekday use	Weekday SOL	0.02 (0.01, 0.03)	0.05 (0.01, 0.10)	0.07 (0.02, 0.11)	25.7
	Weekend use	Weekend SOL	0.03 (0.02, 0.04)	0.06 (0.01, 0.11)	0.09 (0.04, 0.14)	33.1
	Total use	Sleep disturbance	0.01 (0.00, 0.02)	0.08 (0.03, 0.13)	0.09 (0.04, 0.14)	11.5
	Total use	Sleep problems	0.05 (0.03, 0.06)	0.05 (− 0.01, 0.10)	0.09 (0.04, 0.14)	49.6
Anxiety	Weekday use	Insufficient weekday sleep	0.01 (0.00, 0.02)	0.07 (0.02, 0.12)	0.08 (0.03, 0.12)	11.1
	Weekend use	Insufficient weekend sleep	0.01 (− 0.00, 0.02)	0.09 (0.04, 0.14)	0.10 (0.05, 0.15)	–
	Weekday use	Weekday SOL	0.02 (0.01, 0.02)	0.06 (0.02, 0.11)	0.08 (0.03, 0.13)	19.7
	Weekend use	Weekend SOL	0.02 (0.01, 0.04)	0.08 (0.03, 0.13)	0.10 (0.05, 0.15)	23.5
	Total use	Sleep disturbance	0.01 (0.00, 0.01)	0.09 (0.04, 0.15)	0.10 (0.05, 0.15)	6.8
	Total use	Sleep problems	0.03 (0.02, 0.05)	0.07 (0.02, 0.13)	0.10 (0.05, 0.16)	32.4

*SNS *social networking site, *SOL *sleep onset latency, *IQR *interquartile range

Adjusted for age, gender, ethnicity, parental socioeconomic status, school type, and baseline score of Strengths and Difficulties Questionnaire internalising subscale

Depressive and anxiety symptom severity levels (4 levels): no or minimal, mild, moderate, and moderately severe or severe

Sleep problems included all five sleep indices to investigate their joint mediation effects

Table 5 shows that all individual sleep measures partly mediated the associations between SNS use and clinically significant depressive symptoms (mediation proportion ranged between 8.0% and 24.9%), although the mediation effects of sleep disturbance were less marked. SOL, but not insufficient sleep or sleep disturbance, partly mediated the associations between SNS use and clinically significant anxiety symptoms. Combined, sleep problems explained 37.6% and 26.0% of the associations between total SNS use and clinically significant depressive and anxiety symptoms, respectively.

**Table 5:** Mediation effects of sleep on the associations between SNS use across all devices and clinically significant depressive (*n* = 2316) and anxiety symptoms (*n* = 2350)

Clinically significant symptoms	SNS use (per IQR increase)	Sleep measures (mediator)	Indirect effect *b* (95% CI)	Direct effect *b* (95% CI)	Total effect *b* (95% CI)	Mediation proportion (%)
Depressive	Weekday use	Insufficient weekday sleep	0.02 (0.01, 0.03)	0.10 (0.04, 0.16)	0.12 (0.06, 0.17)	15.2
	Weekend use	Insufficient weekend sleep	0.02 (0.00, 0.03)	0.12 (0.06, 0.19)	0.14 (0.08, 0.21)	13.5
	Weekday use	Weekday SOL	0.02 (0.01, 0.03)	0.10 (0.04, 0.15)	0.12 (0.06, 0.17)	15.2
	Weekend use	Weekend SOL	0.04 (0.02, 0.05)	0.11 (0.04, 0.17)	0.14 (0.08, 0.20)	24.9
	Total use	Sleep disturbance	0.01 (0.00, 0.02)	0.13 (0.07, 0.20)	0.15 (0.08, 0.21)	8.0
	Total use	Sleep problems	0.06 (0.03, 0.08)	0.09 (0.03, 0.16)	0.15 (0.08, 0.22)	37.6
Anxiety	Weekday use	Insufficient weekday sleep	0.01 (− 0.00, 0.02)	0.10 (0.04, 0.15)	0.10 (0.05, 0.16)	–
	Weekend use	Insufficient weekend sleep	0.01 (− 0.00, 0.02)	0.10 (0.03, 0.17)	0.11 (0.05, 0.18)	–
	Weekday use	Weekday SOL	0.02 (0.01, 0.03)	0.09 (0.03, 0.15)	0.10 (0.05, 0.16)	14.8
	Weekend use	Weekend SOL	0.02 (0.01, 0.04)	0.09 (0.02, 0.16)	0.11 (0.05, 0.18)	20.7
	Total use	Sleep disturbance	0.01 (− 0.00, 0.01)	0.12 (0.05, 0.18)	0.12 (0.06, 0.19)	–
	Total use	Sleep problems	0.03 (0.01, 0.06)	0.09 (0.02, 0.16)	0.13 (0.06, 0.19)	26.0

*SNS *social networking site, *SOL *sleep onset latency, *IQR *interquartile range

Adjusted for age, gender, ethnicity, parental socioeconomic status, school type, and baseline score of Strengths and Difficulties Questionnaire internalising subscale

Sleep problems included all five sleep indices to investigate their joint mediation effects

SA1 showed that all the above associations remained evident after excluding participants with baseline internalising difficulties (Additional file 1: Tables S2 and S3). In addition, SNS weekend use for more than 3 h/day was associated with higher depressive symptom severity. SA2 showed that the pattern of associations between SNS use and depressive and anxiety symptoms remained similar after additional adjustment for substance use variables (Additional file 1: Tables S4 and S5). Multiple imputation of missing data on covariates did not alter these conclusions (SA3, Additional file 1: Tables S6 and S7). SA4 showed that higher SNS use specifically on mobile phones was also associated with greater depressive and anxiety symptoms. Compared to SNS use on other devices, the associations between SNS use on mobile phones for more than 3 h/day and outcomes were generally stronger (Additional file 1: Tables S8 and S9).

## Discussion

Our study has shown that SNS use exceeding 3 h per day at age 11–12 years was associated with greater depressive and anxiety symptoms at age 13–15 years after controlling for potential confounders and school clustering effects. Sleep problems partly mediated the associations between SNS use and subsequent depressive and anxiety symptoms. The associations between SNS use and depressive symptom severity were present in girls, but not boys. We also found that SNS use on mobile phones for more than 3 h/day had stronger associations with depressive and anxiety symptoms compared to use on other devices.

We focus here on comparing our findings with previous longitudinal studies in adolescents examining SNS use duration and depressive and anxiety symptoms, given the large number of cross-sectional studies on this topic. Our findings are in line with studies (sample size between 763 and 12,035) conducted in Norway, Canada, Iceland, Israel, and India, which showed that longer duration of SNS use predicts greater depressive or anxiety symptoms [17, 37–40]. We add to the evidence base by including more comprehensive SNS use measurements and conducting mediation analyses to increase the strength of causal inference underlying the associations between SNS use and depressive and anxiety symptoms. We also add to the wider and growing evidence base that SNS use may have harmful effects on sleep which impacts depressive and anxiety symptoms. In the same cohort, we found that SNS use at 13–15 years was associated with depressive and anxiety symptoms cross-sectionally before the COVID-19 pandemic, but was not associated with developing clinically significant depressive or anxiety symptoms during the pandemic when they were 16–18 years [41]. Social networks established before the pandemic might have mitigated the impacts of social isolation during lockdown on mental health. Our findings contrast with two US studies, which did not find associations between SNS use duration and depressive or anxiety symptoms [16, 42]. One study (*n* = 9538) has participants with much lower social media use (mean 0.1 h/day), which is unlikely to have mental health impacts [16]. One study has a small sample size (*n* = 487), which might limit the power to detect significant associations [42]. In addition, data of this study were collected between 2007 and 2009, which do not reflect the digital environment one decade later.

It is noteworthy that only SNS use exceeding 3 h per day appeared to significantly increase the risk of depressive and anxiety symptoms, suggesting a potential threshold effect. This finding adds to previous longitudinal studies on SNS use and depressive and anxiety symptoms, which only reported linear associations without explicitly investigating this threshold effect. Further research should explore whether qualitative factors are present among high SNS users, such as maladaptive SNS use, which may underlie the adverse mental health impacts.

To our knowledge, only two studies have investigated the potential underlying mechanisms using mediation analysis [43, 44]. Cyberbullying and displacement of sleep partly mediated the associations between SNS use and anxiety in girls [43], whilst co-rumination partly mediated the associations between SNS use and anxiety symptoms, but not depressive symptoms [44]. However, both studies only considered SNS use frequency, without considering the duration, which is not sufficient to quantify exposure. One study only investigated weekend SNS use [44].

We considered sleep variables as potential mediators and found that insufficient sleep duration mediated the associations between SNS use and depressive symptoms, indicating that the potential harmful effects of SNS use on depressive symptoms might be partly driven by sleep deprivation. The proportion of mediation by insufficient sleep was slightly higher for weekday SNS use. Most schools restricted the use of mobile phones and other wireless devices during school time when data were collected, and participants needed to wake up early to attend school. Therefore, a prime time during which participants can use SNS on weekdays is during the evenings/nights, which could result in direct displacement of sleep time. We found that SOL partly mediated the associations between SNS use and depressive and anxiety symptoms, possibly explained by puberty-related circadian delay, psychological and physiological arousal from emotionally salient content before sleep, as well as melatonin suppression caused by screen light emission at bedtime [45–47]. The proportion of mediation was slightly higher for weekend SNS use, possibly due to the greater nighttime SNS use on weekends compared to weekdays, which could result in longer weekend SOL [48]. Compared to insufficient sleep and SOL, the role of sleep disturbance in explaining the associations between SNS use and depressive and anxiety symptoms was less pronounced but still significant. This is possibly attributed to sleep interruption due to incoming alerts and anxiety about missing out on new content from SNS [49]. The stronger associations of SNS use on mobile phones than other devices with depressive and anxiety symptoms may also be driven by the greater potential to disrupt sleep via mobile phones.

Notably, we found marked associations between SNS use and depressive and anxiety symptoms independent of sleep (i.e. the direct effect), indicating that underlying mechanisms may extend beyond sleep problems. High engagement in SNS may also displace physical activity. Evidence from randomised controlled trials has shown that physical activity has a small but significant beneficial effect on mental health in adolescents in terms of reduction of depressive and anxiety symptoms, as well as improvement of self-esteem [50]. Also, mental health vulnerability is reflected by the development of negative emotional biases, impaired executive control, and heightened emotional reactivity, which may predispose to both maladaptive SNS use and the onset of mental illness [51].

We found a stronger association between SNS use and depressive symptom severity in girls, in line with other studies [10, 15]. Girls show more emotional responses to SNS use, which might predispose them to depressive symptoms [52]. It is also possible that girls are more likely to experience online harassment, cyberbullying, and dissatisfaction with body image, which may lead to feelings of shame and contribute to developing depressive symptoms [10]. We did not observe gender differences in associations between SNS use and anxiety symptoms, suggesting that gender-specific mechanisms might not be at play for anxiety in relation to SNS use.

Our study has strengths and weaknesses. The data come from a large-scale adolescent cohort study with detailed information on SNS use, sleep, and mental health measures. This cohort is representative of school-aged children across Greater London. We were able to distinguish weekday and weekend SNS use as well as the varied mediation effects of sleep. The longitudinal study design enables the investigation of the temporal sequence of SNS use and depressive and anxiety symptoms. However, our study has some limitations. First, we only measured overall SNS use without collecting data on usage on different social network platforms. However, at the time of SCAMP baseline data collection (2014–2016), the range of social network platforms was considerably narrower than it is today. Additionally, children now have access to mobile phones and SNS at earlier ages than when the baseline data were collected [53]. Further research using more recent and nuanced data is warranted to investigate the mental health impacts of SNS use, which might be more pronounced in contemporary adolescents. Second, the use of self-reported questionnaires to collect information on SNS use and sleep in our study is open to recall bias. Data collected from mobile phone applications to capture time spent on different social network platforms and wearable devices (e.g. actigraphy) to capture sleep are needed to minimise biases associated with self-report. Third, we only investigated the associations between baseline SNS use and follow-up depressive and anxiety symptoms to establish a clear temporal sequence. However, given that SNS use increased at follow-up, our findings may underestimate the strength of the associations between SNS use and depressive and anxiety symptoms. Repeated measures of SNS use and depressive and anxiety symptoms would enhance measurement stability and demonstrate time effects and trends, ultimately providing a better understanding of the underlying explanatory mechanisms. Fourth, there is the possibility of some residual confounding, as we could not adjust directly for family mental health issues, bullying victimisation, or peer influence, which may associate with both exposure and outcome. However, we did adjust for baseline internalising difficulties, which comprises the peer relationship problems scale with a question on bullying. Moreover, we controlled for school clustering effects which also capture peer-related effects within schools. Fifth, we investigated sleep measures at baseline as mediators of the longitudinal association between SNS use and depressive and anxiety symptoms. However, considering the bidirectional associations between sleep and mental health problems previously found in the same cohort [23], we cannot rule out the possibility of reverse causation given that sleep problems may lead to more SNS use. Sixth, the geographic distribution of SCAMP participants may limit the generalisability of our findings to adolescents beyond the Greater London area.

Our findings have several public health implications. Our study indicates the potential adverse effects of high SNS use at ages 11–12 years on depressive and anxiety symptoms at ages 13–15 years. This highlights the necessity for early identification of high-risk groups and timely prevention strategies. Girls might be more vulnerable to depressive symptom severity in relation to SNS use; therefore, public health messaging and intervention design tackling high SNS use and depressive symptoms should be gender specific. In addition, insufficient sleep and SOL may be targets for early intervention to partly mitigate the risk of high SNS use on the development of depressive and anxiety symptoms.

## Conclusions

Our study showed that SNS use exceeding 3 h per day was associated with higher risks of depressive and anxiety symptoms in adolescents, suggesting that high SNS users should be the targeted group for mental health interventions. Insufficient sleep and SOL partly mediated the associations. Future research should have more detailed measurements of SNS such as social media content type and user characteristics to enhance the understanding of the impact of SNS on mental health. In addition, a more detailed and diverse set of mental health outcomes could be drawn by combining self-reports with complementary clinical data such as health record linkage, electronic health records, and structured interviews. Given that high social media use is already evident by the start of secondary school and contributes to later mental health risks, our findings may inform the development of targets for behavioural interventions and mechanistic research (e.g. early secondary school curricula incorporating digital literacy and sleep hygiene education) to protect mental health.

## Supplementary Information

Additional file 1. Fig. S1 Correlations between SNS use across all devices and PHQ-9 and GAD-7 scores. Table S1 Descriptive statistics between the analytical sample and participants who only participated in the baseline assessment. Table S2 Associations between baseline SNS use across all devices and depressive and anxiety symptom severity levels at 2-year follow-up by excluding participants with internalising difficulties at baseline. Table S3 Associations between baseline SNS use across all devices and clinically significant depressive and anxiety symptoms at 2-year follow-up by excluding participants with internalising difficulties at baseline. Table S4 Associations between baseline SNS use across all devices and depressive and anxiety symptom severity levels at 2-year follow-up after additionally adjusting for substance use at baseline. Table S5 Associations between baseline SNS use across all devices and clinically significant depressive and anxiety symptoms at 2-year follow-up after additionally adjusting for substance use at baseline. Table S6 Associations between baseline SNS use across all devices and depressive and anxiety symptom severity levels at 2-year follow-up using multiple imputation. Table S7 Associations between baseline SNS use across all devices and clinically significant depressive and anxiety symptoms at 2-year follow-up using multiple imputation. Table S8 Associations of baseline SNS use on mobile phones and other devices with depressive and anxiety symptom severity levels at 2-year follow-up. Table S9 Associations of baseline SNS use on mobile phones and other devices with clinically significant depressive and anxiety symptoms at 2-year follow-up

## Data Availability

According to the participant consent, access to individual-level SCAMP data needs to be approved by the SCAMP Data Access Committee (https://scampstudy.org/get-involved/opportunities-for-researchers/). Application should be made to scamp@imperial.ac.uk. The data dictionary is available on request to the corresponding author.

## Abbreviations

GAD-7: 7-Item Generalised Anxiety Disorder scale
IQR: Interquartile range
NS-SEC: National Statistics Socio-economic classification
OR: Odds ratio
PHQ-9: 9-Item Patient Health Questionnaire
SCAMP: Study of Cognition, Adolescents, and Mobile Phones
SNS: Social network site
SOL: Sleep onset latency
SES: Socioeconomic status
SDQ: Strengths and Difficulties Questionnaire
