# Supplementary material for: Social networking site use, depressive and anxiety symptoms in adolescents: evidence from a longitudinal cohort study (SCAMP)
Source: BMC Med. 2026 Feb 3;24:139. doi: 10.1186/s12916-026-04667-5 (PMC12958652; doi:10.1186/s12916-026-04667-5)
Supplement: Supplementary file 1 — Additional file 1. Fig. S1 Correlations between SNS use across all devices and PHQ-9 and GAD-7 scores. Table S1 Descriptive statistics between the analytical sample and participants who only participated in the baseline assessment. Table S2 Associations between baseline SNS use across all devices and depressive and anxiety symptom severity levels at 2-year follow-up by excluding participants with internalising difficulties at baseline. Table S3 Associations between baseline SNS use across all devices and clinically significant depressive and anxiety symptoms at 2-year follow-up by excluding participants with internalising difficulties at baseline. Table S4 Associations between baseline SNS use across all devices and depressive and anxiety symptom severity levels at 2-year follow-up after additionally adjusting for substance use at baseline. Table S5 Associations between baseline SNS use across all devices and clinically significant depressive and anxiety symptoms at 2-year follow-up after additionally adjusting for substance use at baseline. Table S6 Associations between baseline SNS use across all devices and depressive and anxiety symptom severity levels at 2-year follow-up using multiple imputation. Table S7 Associations between baseline SNS use across all devices and clinically significant depressive and anxiety symptoms at 2-year follow-up using multiple imputation. Table S8 Associations of baseline SNS use on mobile phones and other devices with depressive and anxiety symptom severity levels at 2-year follow-up. Table S9 Associations of baseline SNS use on mobile phones and other devices with clinically significant depressive and anxiety symptoms at 2-year follow-up [file 12916_2026_4667_MOESM1_ESM.docx]

Fig S1 Correlations between SNS use across all devices and PHQ-9 and GAD-7 scores


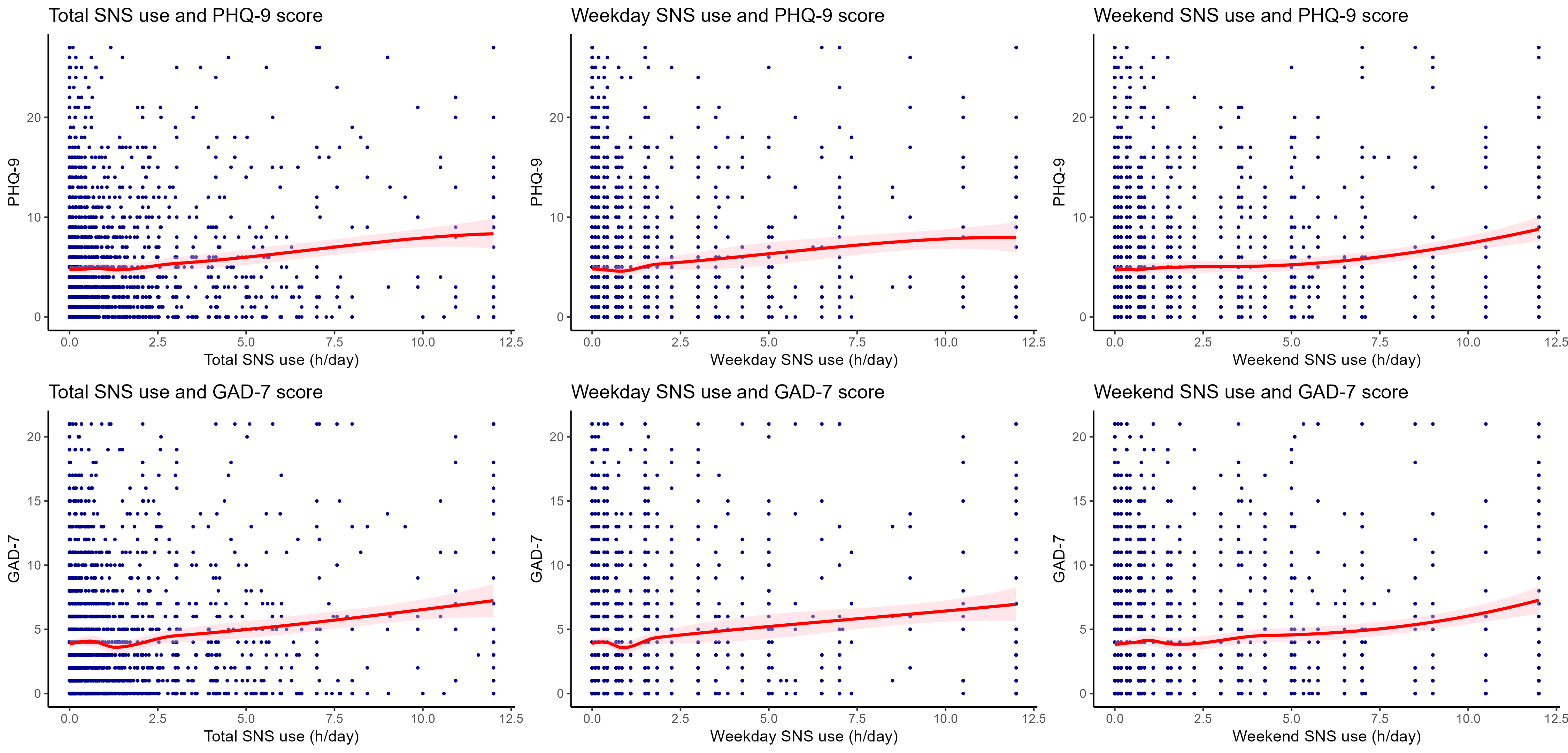


Table S1 Descriptive statistics between the analytical sample and participants who only participated in the baseline assessment

| **Socio-demographic variables** | Depressive (n=2316) | Anxiety (n=2350) | Baseline only (n=1954) |
| --- | --- | --- | --- |
| Age (years) at baseline, mean (SD) | 11.59 (0.47) | 11.59 (0.47) | 11.68 (0.49) |
| Gender, n (%) |  |  |  |
| Male | 1044 (45.1) | 1058 (45.0) | 1071 (54.8) |
| Female | 1272 (54.9) | 1292 (55.0) | 883 (45.2) |
| Ethnicity, n (%) |  |  |  |
| White | 993 (42.9) | 1006 (42.8) | 852 (43.6) |
| Black | 244 (10.5) | 250 (10.6) | 236 (12.1) |
| Asian | 774 (33.4) | 781 (33.2) | 562 (28.8) |
| Mixed/others | 305 (13.2) | 313 (13.3) | 304 (15.6) |
| Parental socioeconomic status^a^, n (%) |  |  |  |
| Managerial/professional occupations | 1373 (59.3) | 1389 (59.1) | 1086 (55.6) |
| Intermediate occupations | 503 (21.7) | 511 (21.7) | 467 (23.9) |
| Routine and manual occupations | 440 (19.0) | 450 (19.2) | 401 (20.5) |
| Type of school, n (%) |  |  |  |
| Independent | 684 (29.5) | 687 (29.2) | 341 (17.5) |
| State | 1632 (70.5) | 1663 (70.8) | 1613 (82.5) |
| **Exposures (baseline, across all devices)** |  |  |  |
| Total SNS use (h), median (IQR) | 0.34 (0.03, 1.24) | 0.34 (0.03, 1.23) | 0.55 (0.09, 2.07) |
| Weekday average SNS use (h), median (IQR) | 0.34 (0, 1.09) | 0.34 (0, 1.09) | 0.43 (0.09, 1.59) |
| Weekend average SNS use (h), median (IQR) | 0.43 (0.05, 1.5) | 0.43 (0.09, 1.5) | 0.75 (0.09, 3) |

IQR: interquartile range; SNS: social networking site

Difference between depressive analytical sample and baseline only sample: all P values < 0.05 except parental socioeconomic status

Difference between anxiety analytical sample and baseline only sample: all P values < 0.05 except parental socioeconomic status

a: Parental socioeconomic status was derived based on National Statistics Socio-economic classification of occupation

Table S2 Associations between baseline SNS use across all devices and depressive and anxiety symptom severity levels at 2-year follow-up by excluding participants with internalising difficulties at baseline, using ordinal logistic regression

| Symptom severity | SNS use | Daily average duration | N | Adjusted model  OR (95% CI) |
| --- | --- | --- | --- | --- |
| Depressive | Total use | 0-30min | 1268 | 1 |
|  |  | 31-59min | 295 | 1.17 (0.90, 1.52) |
|  |  | 1-2h | 352 | 1.23 (0.96, 1.57) |
|  |  | 3h+ | 235 | **1.52 (1.14, 2.04)** |
|  | Per IQR increase | 1.31h | 2150 | **1.12 (1.05, 1.18)** |
|  | Weekday use | 0-30min | 1386 | 1 |
|  |  | 31-59min | 241 | 1.25 (0.94, 1.65) |
|  |  | 1-2h | 311 | 1.26 (0.98, 1.62) |
|  |  | 3h+ | 212 | **1.51 (1.11, 2.04)** |
|  | Per IQR increase | 1.09h | 2150 | **1.09 (1.04, 1.14)** |
|  | Weekend use | 0-30min | 1171 | 1 |
|  |  | 31-59min | 264 | 0.92 (0.69, 1.23) |
|  |  | 1-2h | 336 | **1.28 (1.00, 1.63)** |
|  |  | 3h+ | 379 | **1.29 (1.01, 1.65)** |
|  | Per IQR increase | 1.5h | 2150 | **1.12 (1.06, 1.18)** |
| Anxiety | Total use | 0-30min | 1284 | 1 |
|  |  | 31-59min | 299 | 0.99 (0.75, 1.31) |
|  |  | 1-2h | 356 | 1.14 (0.88, 1.47) |
|  |  | 3h+ | 239 | **1.51 (1.12, 2.03)** |
|  | Per IQR increase | 1.31h | 2178 | **1.13 (1.06, 1.19)** |
|  | Weekday use | 0-30min | 1405 | 1 |
|  |  | 31-59min | 244 | 0.84 (0.62, 1.14) |
|  |  | 1-2h | 313 | 1.13 (0.86, 1.48) |
|  |  | 3h+ | 216 | **1.47 (1.08, 2.00)** |
|  | Per IQR increase | 1.09h | 2178 | **1.10 (1.04, 1.16)** |
|  | Weekend use | 0-30min | 1183 | 1 |
|  |  | 31-59min | 271 | 0.96 (0.71, 1.30) |
|  |  | 1-2h | 340 | 1.00 (0.77, 1.31) |
|  |  | 3h+ | 384 | **1.39 (1.09, 1.79)** |
|  | Per IQR increase | 1.5h | 2178 | **1.12 (1.06, 1.19)** |

SNS: social networking site; OR: odds ratio; IQR: interquartile range

Exclude participants with internalising difficulties at baseline (n=263)

Adjusted for age, gender, ethnicity, parental socioeconomic status, baseline score of Strengths and Difficulties Questionnaire internalising subscale, and school clustering effect.

Depressive and anxiety symptom severity levels (4 levels): no or minimal, mild, moderate, and moderately severe or severe

OR in ordinal logistic regression reflects the change in the odds of being in a higher category of the outcome variable (e.g., depressive and anxiety symptom severity levels) associated with a one-unit increase in the exposure variable (e.g., total SNS use).

Table S3 Associations between baseline SNS use across all devices and clinically significant depressive and anxiety symptoms at 2-year follow-up by excluding participants with internalising difficulties at baseline

| Clinically significant symptoms | SNS use | Daily average duration | N | Adjusted model  OR (95% CI) |
| --- | --- | --- | --- | --- |
| Depressive | Total use | 0-30min | 1268 | 1 |
|  |  | 31-59min | 295 | 0.97 (0.64, 1.47) |
|  |  | 1-2h | 352 | 1.24 (0.86, 1.78) |
|  |  | 3h+ | 235 | **1.85 (1.25, 2.73)** |
|  | Per IQR increase | 1.31h | 2150 | **1.19 (1.11, 1.28)** |
|  | Weekday use | 0-30min | 1386 | 1 |
|  |  | 31-59min | 241 | 1.17 (0.76, 1.80) |
|  |  | 1-2h | 311 | 1.24 (0.84, 1.82) |
|  |  | 3h+ | 212 | **2.05 (1.38, 3.04)** |
|  | Per IQR increase | 1.09h | 2150 | **1.15 (1.08, 1.22)** |
|  | Weekend use | 0-30min | 1171 | 1 |
|  |  | 31-59min | 264 | 1.06 (0.69, 1.63) |
|  |  | 1-2h | 336 | 1.06 (0.72, 1.57) |
|  |  | 3h+ | 379 | **1.64 (1.17, 2.29)** |
|  | Per IQR increase | 1.5h | 2150 | **1.19 (1.11, 1.27)** |
| Anxiety | Total use | 0-30min | 1284 | 1 |
|  |  | 31-59min | 299 | 1.06 (0.70, 1.62) |
|  |  | 1-2h | 356 | 1.15 (0.78, 1.70) |
|  |  | 3h+ | 239 | **1.86 (1.24, 2.79)** |
|  | Per IQR increase | 1.31h | 2178 | **1.16 (1.08, 1.25)** |
|  | Weekday use | 0-30min | 1405 | 1 |
|  |  | 31-59min | 244 | 0.67 (0.40, 1.14) |
|  |  | 1-2h | 313 | 1.33 (0.90, 1.96) |
|  |  | 3h+ | 216 | **1.76 (1.16, 2.66)** |
|  | Per IQR increase | 1.09h | 2178 | **1.14 (1.07, 1.21)** |
|  | Weekend use | 0-30min | 1183 | 1 |
|  |  | 31-59min | 271 | 1.05 (0.67, 1.63) |
|  |  | 1-2h | 340 | 0.94 (0.62, 1.42) |
|  |  | 3h+ | 384 | **1.52 (1.07, 2.17)** |
|  | Per IQR increase | 1.5h | 2178 | **1.14 (1.06, 1.23)** |

SNS: social networking site; OR: odds ratio; IQR: interquartile range

Exclude participants with internalising difficulties at baseline (n=263)

Adjusted for age, gender, ethnicity, parental socioeconomic status, baseline score of Strengths and Difficulties Questionnaire internalising subscale, and school clustering effect.

Table S4 Associations between baseline SNS use across all devices and depressive and anxiety symptom severity levels at 2-year follow-up after additionally adjusting for substance use at baseline, using ordinal logistic regression

| Symptom severity | SNS use | Daily average duration | N | Adjusted model  OR (95% CI) |
| --- | --- | --- | --- | --- |
| Depressive | Total use | 0-30min | 1346 | 1 |
|  |  | 31-59min | 311 | 1.20 (0.94, 1.55) |
|  |  | 1-2h | 385 | 1.11 (0.88, 1.41) |
|  |  | 3h+ | 274 | **1.44 (1.10, 1.90)** |
|  | Per IQR increase | 1.31h | 2316 | **1.09 (1.04, 1.15)** |
|  | Weekday use | 0-30min | 1473 | 1 |
|  |  | 31-59min | 250 | 1.19 (0.91, 1.56) |
|  |  | 1-2h | 346 | 1.12 (0.88, 1.43) |
|  |  | 3h+ | 247 | **1.42 (1.07, 1.88)** |
|  | Per IQR increase | 1.09h | 2316 | **1.07 (1.03, 1.12)** |
|  | Weekend use | 0-30min | 1241 | 1 |
|  |  | 31-59min | 276 | 0.88 (0.66, 1.16) |
|  |  | 1-2h | 368 | 1.24 (0.98, 1.57) |
|  |  | 3h+ | 431 | 1.20 (0.95, 1.52) |
|  | Per IQR increase | 1.5h | 2316 | **1.10 (1.04, 1.15)** |
| Anxiety | Total use | 0-30min | 1365 | 1 |
|  |  | 31-59min | 316 | 1.04 (0.80, 1.36) |
|  |  | 1-2h | 390 | 1.07 (0.84, 1.37) |
|  |  | 3h+ | 279 | **1.39 (1.06, 1.83)** |
|  | Per IQR increase | 1.31h | 2350 | **1.10 (1.05, 1.16)** |
|  | Weekday use | 0-30min | 1497 | 1 |
|  |  | 31-59min | 253 | 0.82 (0.60, 1.10) |
|  |  | 1-2h | 348 | 1.08 (0.84, 1.39) |
|  |  | 3h+ | 252 | **1.33 (1.00, 1.76)** |
|  | Per IQR increase | 1.09h | 2350 | **1.08 (1.03, 1.13)** |
|  | Weekend use | 0-30min | 1254 | 1 |
|  |  | 31-59min | 285 | 0.95 (0.71, 1.26) |
|  |  | 1-2h | 373 | 0.99 (0.77, 1.27) |
|  |  | 3h+ | 438 | **1.30 (1.02, 1.64)** |
|  | Per IQR increase | 1.5h | 2350 | **1.10 (1.05, 1.16)** |

SNS: social networking site; OR: odds ratio; IQR: interquartile range

Adjusted for age, gender, ethnicity, parental socioeconomic status, baseline score of Strengths and Difficulties Questionnaire internalising subscale, alcohol use, tobacco smoking, marijuana use, and school clustering effect.

Depressive and anxiety symptom severity levels (4 levels): no or minimal, mild, moderate, and moderately severe or severe

OR in ordinal logistic regression reflects the change in the odds of being in a higher category of the outcome variable (e.g., depressive and anxiety symptom severity levels) associated with a one-unit increase in the exposure variable (e.g., total SNS use).

Table S5 Associations between baseline SNS use across all devices and clinically significant depressive and anxiety symptoms at 2-year follow-up after additionally adjusting for substance use at baseline

| Clinically significant symptoms | SNS use | Daily average duration | N | Adjusted model  OR (95% CI) |
| --- | --- | --- | --- | --- |
| Depressive | Total use | 0-30min | 1346 | 1 |
|  |  | 31-59min | 311 | 0.99 (0.67, 1.45) |
|  |  | 1-2h | 385 | 1.06 (0.75, 1.50) |
|  |  | 3h+ | 274 | **1.61 (1.12, 2.31)** |
|  | Per IQR increase | 1.31h | 2316 | **1.15 (1.08, 1.23)** |
|  | Weekday use | 0-30min | 1473 | 1 |
|  |  | 31-59min | 250 | 1.05 (0.69, 1.59) |
|  |  | 1-2h | 346 | 1.03 (0.71, 1.48) |
|  |  | 3h+ | 247 | **1.77 (1.23, 2.55)** |
|  | Per IQR increase | 1.09h | 2316 | **1.12 (1.06, 1.18)** |
|  | Weekend use | 0-30min | 1241 | 1 |
|  |  | 31-59min | 276 | 0.95 (0.62, 1.43) |
|  |  | 1-2h | 368 | 0.99 (0.69, 1.42) |
|  |  | 3h+ | 431 | **1.43 (1.05, 1.96)** |
|  | Per IQR increase | 1.5h | 2316 | **1.15 (1.08, 1.22)** |
| Anxiety | Total use | 0-30min | 1365 | 1 |
|  |  | 31-59min | 316 | 1.04 (0.71, 1.54) |
|  |  | 1-2h | 390 | 1.06 (0.74, 1.52) |
|  |  | 3h+ | 279 | **1.58 (1.09, 2.28)** |
|  | Per IQR increase | 1.31h | 2350 | **1.13 (1.06, 1.21)** |
|  | Weekday use | 0-30min | 1497 | 1 |
|  |  | 31-59min | 253 | 0.65 (0.39, 1.06) |
|  |  | 1-2h | 348 | 1.21 (0.84, 1.73) |
|  |  | 3h+ | 252 | **1.50 (1.03, 2.19)** |
|  | Per IQR increase | 1.09h | 2350 | **1.11 (1.05, 1.17)** |
|  | Weekend use | 0-30min | 1254 | 1 |
|  |  | 31-59min | 285 | 1.00 (0.66, 1.52) |
|  |  | 1-2h | 373 | 0.86 (0.59, 1.26) |
|  |  | 3h+ | 438 | 1.36 (0.98, 1.88) |
|  | Per IQR increase | 1.5h | 2350 | **1.12 (1.05, 1.19)** |

SNS: social networking site; OR: odds ratio; IQR: interquartile range

Adjusted for age, gender, ethnicity, parental socioeconomic status, baseline score of Strengths and Difficulties Questionnaire internalising subscale, alcohol use, tobacco smoking, marijuana use, and school clustering effect.

Table S6 Associations between baseline SNS use across all devices and depressive and anxiety symptom severity levels at 2-year follow-up using multiple imputation

| Symptom severity | SNS use | Daily average duration | Adjusted model  OR (95% CI) |
| --- | --- | --- | --- |
| Depressive | Total use | 0-30min | 1 |
|  |  | 31-59min | 1.14 (0.89, 1.45) |
|  |  | 1-2h | 1.16 (0.92, 1.45) |
|  |  | 3h+ | **1.42 (1.10, 1.83)** |
|  | Per IQR increase | 1.31h | **1.07 (1.02, 1.13)** |
|  | Weekday use | 0-30min | 1 |
|  |  | 31-59min | 1.14 (0.88, 1.48) |
|  |  | 1-2h | 1.19 (0.94, 1.50) |
|  |  | 3h+ | **1.38 (1.06, 1.79)** |
|  | Per IQR increase | 1.09h | **1.06 (1.01, 1.10)** |
|  | Weekend use | 0-30min | 1 |
|  |  | 31-59min | 0.89 (0.68, 1.17) |
|  |  | 1-2h | 1.23 (0.99, 1.54) |
|  |  | 3h+ | **1.25 (1.00, 1.55)** |
|  | Per IQR increase | 1.5h | **1.08 (1.03, 1.14)** |
| Anxiety | Total use | 0-30min | 1 |
|  |  | 31-59min | 1.01 (0.78, 1.31) |
|  |  | 1-2h | 1.09 (0.86, 1.38) |
|  |  | 3h+ | **1.37 (1.06, 1.77)** |
|  | Per IQR increase | 1.31h | **1.09 (1.04, 1.14)** |
|  | Weekday use | 0-30min | 1 |
|  |  | 31-59min | 0.83 (0.62, 1.10) |
|  |  | 1-2h | 1.10 (0.86, 1.40) |
|  |  | 3h+ | **1.32 (1.01, 1.72)** |
|  | Per IQR increase | 1.09h | **1.07 (1.02, 1.11)** |
|  | Weekend use | 0-30min | 1 |
|  |  | 31-59min | 0.99 (0.75, 1.30) |
|  |  | 1-2h | 1.00 (0.79, 1.28) |
|  |  | 3h+ | **1.32 (1.06, 1.65)** |
|  | Per IQR increase | 1.5h | **1.09 (1.04, 1.14)** |

SNS: social networking site; OR: odds ratio; IQR: interquartile range

Adjusted for age, gender, ethnicity, parental socioeconomic status, baseline score of Strengths and Difficulties Questionnaire internalising subscale, and school clustering effect.

Depressive and anxiety symptom severity levels (4 levels): no or minimal, mild, moderate, and moderately severe or severe

OR in ordinal logistic regression reflects the change in the odds of being in a higher category of the outcome variable (e.g., depressive and anxiety symptom severity levels) associated with a one-unit increase in the exposure variable (e.g., total SNS use).

Table S7 Associations between baseline SNS use across all devices and clinically significant depressive and anxiety symptoms at 2-year follow-up using multiple imputation

| Clinically significant symptoms | SNS use | Daily average duration | Adjusted model  OR (95% CI) |
| --- | --- | --- | --- |
| Depressive | Total use | 0-30min | 1 |
|  |  | 31-59min | 0.88 (0.60, 1.28) |
|  |  | 1-2h | 1.13 (0.81, 1.56) |
|  |  | 3h+ | **1.52 (1.08, 2.14)** |
|  | Per IQR increase | 1.31h | **1.12 (1.06, 1.19)** |
|  | Weekday use | 0-30min | 1 |
|  |  | 31-59min | 0.95 (0.63, 1.43) |
|  |  | 1-2h | 1.11 (0.79, 1.57) |
|  |  | 3h+ | **1.71 (1.21, 2.40)** |
|  | Per IQR increase | 1.09h | **1.09 (1.04, 1.15)** |
|  | Weekend use | 0-30min | 1 |
|  |  | 31-59min | 1.00 (0.68, 1.49) |
|  |  | 1-2h | 0.97 (0.69, 1.38) |
|  |  | 3h+ | **1.45 (1.08, 1.96)** |
|  | Per IQR increase | 1.5h | **1.13 (1.06, 1.20)** |
| Anxiety | Total use | 0-30min | 1 |
|  |  | 31-59min | 0.99 (0.68, 1.45) |
|  |  | 1-2h | 1.13 (0.80, 1.59) |
|  |  | 3h+ | **1.57 (1.11, 2.22)** |
|  | Per IQR increase | 1.31h | **1.11 (1.05, 1.18)** |
|  | Weekday use | 0-30min | 1 |
|  |  | 31-59min | 0.64 (0.40, 1.04) |
|  |  | 1-2h | 1.26 (0.89, 1.78) |
|  |  | 3h+ | **1.52 (1.07, 2.16)** |
|  | Per IQR increase | 1.09h | **1.09 (1.04, 1.15)** |
|  | Weekend use | 0-30min | 1 |
|  |  | 31-59min | 1.06 (0.71, 1.57) |
|  |  | 1-2h | 0.86 (0.60, 1.25) |
|  |  | 3h+ | **1.40 (1.03, 1.91)** |
|  | Per IQR increase | 1.5h | **1.11 (1.04, 1.18)** |

SNS: social networking site; OR: odds ratio; IQR: interquartile range

Adjusted for age, gender, ethnicity, parental socioeconomic status, baseline score of Strengths and Difficulties Questionnaire internalising subscale, and school clustering effect.

Table S8 Associations of baseline SNS use on mobile phones and other devices with depressive and anxiety symptom severity levels at 2-year follow-up, using ordinal logistic regression

|  |  |  | SNS use on  mobile phones | | SNS use on  other devices | |
| --- | --- | --- | --- | --- | --- | --- |
| Symptom severity | SNS use | Daily average  duration | N | Adjusted model  OR (95% CI) | N | Adjusted model  OR (95% CI) |
| Depressive | Total use | 0-30min | 1689 | 1 | 1811 | 1 |
|  |  | 31-59min | 240 | 1.04 (0.77, 1.39) | 209 | 1.09 (0.80, 1.48) |
|  |  | 1-2h | 243 | 1.11 (0.83, 1.50) | 184 | 0.94 (0.67, 1.33) |
|  |  | 3h+ | 144 | **1.59 (1.06, 2.39)** | 112 | 1.39 (0.88, 2.19) |
|  | Weekday use | 0-30min | 1744 | 1 | 1854 | 1 |
|  |  | 31-59min | 224 | 1.08 (0.79, 1.46) | 207 | 1.09 (0.80, 1.47) |
|  |  | 1-2h | 191 | 1.17 (0.84, 1.61) | 136 | 0.93 (0.63, 1.37) |
|  |  | 3h+ | 157 | **1.60 (1.08, 2.38)** | 119 | 1.15 (0.74, 1.80) |
|  | Weekend use | 0-30min | 1543 | 1 | 1682 | 1 |
|  |  | 31-59min | 263 | **1.41 (1.07, 1.84)** | 234 | 0.83 (0.62, 1.11) |
|  |  | 1-2h | 217 | 1.05 (0.77, 1.44) | 193 | 0.79 (0.57, 1.11) |
|  |  | 3h+ | 293 | **1.54 (1.14, 2.08)** | 207 | 1.25 (0.89, 1.75) |
| Anxiety | Total use | 0-30min | 1713 | 1 | 1840 | 1 |
|  |  | 31-59min | 242 | 1.10 (0.81, 1.49) | 210 | 0.88 (0.63, 1.23) |
|  |  | 1-2h | 248 | 1.15 (0.85, 1.56) | 187 | 0.88 (0.62, 1.26) |
|  |  | 3h+ | 147 | **1.74 (1.15, 2.64)** | 113 | 1.31 (0.83, 2.07) |
|  | Weekday use | 0-30min | 1770 | 1 | 1883 | 1 |
|  |  | 31-59min | 226 | 1.09 (0.79, 1.49) | 208 | 0.88 (0.63, 1.23) |
|  |  | 1-2h | 194 | 1.29 (0.93, 1.79) | 139 | 0.94 (0.64, 1.40) |
|  |  | 3h+ | 160 | **1.61 (1.08, 2.40)** | 120 | 1.17 (0.74, 1.83) |
|  | Weekend use | 0-30min | 1563 | 1 | 1709 | 1 |
|  |  | 31-59min | 268 | 1.32 (0.99, 1.75) | 236 | 0.74 (0.54, 1.01) |
|  |  | 1-2h | 220 | 1.14 (0.83, 1.58) | 197 | 0.69 (0.48, 0.98) |
|  |  | 3h+ | 299 | **1.56 (1.15, 2.12)** | 208 | 1.24 (0.88, 1.74) |

SNS: social networking site; OR: odds ratio; IQR: interquartile range

Adjusted for age, gender, ethnicity, parental socioeconomic status, baseline score of Strengths and Difficulties Questionnaire internalising subscale, and school clustering effect. SNS use on mobile phones and SNS use on other devices were mutually adjusted.

Depressive and anxiety symptom severity levels (4 levels): no or minimal, mild, moderate, and moderately severe or severe

OR in ordinal logistic regression reflects the change in the odds of being in a higher category of the outcome variable (e.g., depressive and anxiety symptom severity levels) associated with a one-unit increase in the exposure variable (e.g., total SNS use).

Table S9 Associations of baseline SNS use on mobile phones and other devices with clinically significant depressive and anxiety symptoms at 2-year follow-up

|  |  |  | SNS use on  mobile phones | | SNS use on  other devices | |
| --- | --- | --- | --- | --- | --- | --- |
| Clinically significant symptoms | SNS use | Daily average  duration | N | Adjusted model  OR (95% CI) | N | Adjusted model  OR (95% CI) |
| Depressive | Total use | 0-30min | 1689 | 1 | 1811 | 1 |
|  |  | 31-59min | 240 | 1.11 (0.71, 1.73) | 209 | 0.79 (0.48, 1.29) |
|  |  | 1-2h | 243 | 1.08 (0.70, 1.65) | 184 | 0.97 (0.60, 1.58) |
|  |  | 3h+ | 144 | **2.03 (1.20, 3.44)** | 112 | 1.68 (0.95, 2.98) |
|  | Weekday use | 0-30min | 1744 | 1 | 1854 | 1 |
|  |  | 31-59min | 224 | 1.12 (0.71, 1.77) | 207 | 0.70 (0.42, 1.18) |
|  |  | 1-2h | 191 | 1.14 (0.72, 1.80) | 136 | 1.12 (0.68, 1.87) |
|  |  | 3h+ | 157 | **1.93 (1.16, 3.20)** | 119 | 1.47 (0.84, 2.59) |
|  | Weekend use | 0-30min | 1543 | 1 | 1682 | 1 |
|  |  | 31-59min | 263 | 1.20 (0.80, 1.79) | 234 | 0.76 (0.49, 1.19) |
|  |  | 1-2h | 217 | 1.05 (0.65, 1.68) | 193 | 0.80 (0.48, 1.32) |
|  |  | 3h+ | 293 | **1.75 (1.17, 2.61)** | 207 | 1.44 (0.92, 2.24) |
| Anxiety | Total use | 0-30min | 1713 | 1 | 1840 | 1 |
|  |  | 31-59min | 242 | 1.06 (0.67, 1.67) | 210 | 1.04 (0.64, 1.69) |
|  |  | 1-2h | 248 | 1.01 (0.65, 1.55) | 187 | 1.24 (0.76, 2.01) |
|  |  | 3h+ | 147 | **1.74 (1.01, 2.99)** | 113 | 1.48 (0.81, 2.71) |
|  | Weekday use | 0-30min | 1770 | 1 | 1883 | 1 |
|  |  | 31-59min | 226 | 1.01 (0.63, 1.63) | 208 | 1.03 (0.63, 1.68) |
|  |  | 1-2h | 194 | 1.21 (0.77, 1.90) | 139 | 1.30 (0.78, 2.18) |
|  |  | 3h+ | 160 | 1.55 (0.92, 2.62) | 120 | 1.44 (0.80, 2.60) |
|  | Weekend use | 0-30min | 1563 | 1 | 1709 | 1 |
|  |  | 31-59min | 268 | 1.18 (0.78, 1.77) | 236 | 0.85 (0.54, 1.32) |
|  |  | 1-2h | 220 | 1.01 (0.62, 1.65) | 197 | 0.73 (0.43, 1.25) |
|  |  | 3h+ | 299 | **1.56 (1.03, 2.37)** | 208 | 1.38 (0.87, 2.19) |

SNS: social networking site; OR: odds ratio; IQR: interquartile range

Adjusted for age, gender, ethnicity, parental socioeconomic status, baseline score of Strengths and Difficulties Questionnaire internalising subscale, and school clustering effect. SNS use on mobile phones and SNS use on other devices were mutually adjusted.
